# Enterprise Imaging Governance: HIMSS-SIIM Collaborative White Paper

**DOI:** 10.1007/s10278-016-9883-z

**Published:** 2016-06-14

**Authors:** Christopher J. Roth, Louis M. Lannum, Carol L. Joseph

**Affiliations:** 1 Duke Health Technology Solutions, Hock Plaza, 2424 Erwin Road, Durham, NC 27705 USA; 2 Department of Radiology, Duke University Hospital, 2301 Erwin Road, Box 3808, Durham, NC 27710 USA; 3 Information Technology Division, Cleveland Clinic, 572 Lake Forest Dr., Bay Village, Ohio, 44140-2513 USA; 4 Ascension Information Services, Clinical Information Services, 4600 Edmundson Road, St. Louis, MO 63134 USA

**Keywords:** Diagnostic Imaging, Digital Image Management, Digital Imaging, Digital Imaging and Communications in Medicine (DICOM), Electronic Medical Record (EMR), Enterprise PACS, Image Data, Image Distribution, Image Display, Imaging Informatics, Multimedia, PACS, PACS Administration, Enterprise Imaging, Governance

## Abstract

Enterprise imaging governance is an emerging need in health enterprises today. This white paper highlights the decision-making body, framework, and process for optimal enterprise imaging governance inclusive of five areas of focus: program governance, technology governance, information governance, clinical governance, and financial governance. It outlines relevant parallels and differences when forming or optimizing imaging governance as compared with other established broad horizontal governance groups, such as for the electronic health record. It is intended for CMIOs and health informatics leaders looking to grow and govern a program to optimally capture, store, index, distribute, view, exchange, and analyze the images of their enterprise.

## Introduction: What Is Enterprise Imaging Governance?

The Institute for Healthcare Improvement (IHI) describes the now familiar Triple Aim of improving the patient experience of care, improving the health of populations, and reducing the per capita cost of health care [1]. Better care coordination and health information technology integration are expected to play a large role in achieving this Triple Aim [2, 3]. Today, often care coordination and health information technology integration are ensured by appropriate governance of electronic health records [4, 5]. Only 218 and 207 references returned with recent PubMed searches of “electronic health record governance” and “electronic medical record governance,” respectively, more than half of which were 2012 citations and sooner [6, 7]. Many of these results contained discussion of the need for governance, rather than the structure and implementation of governance. This would suggest that the implementation, review, and study of organizational governance frameworks and committees are more recent developments in healthcare. Successful governance may be defined where there is active cooperation and approval of clinicians in implementation of clinical systems and may include critical metrics around physician and nurse leaders serving on an institution’s or group’s governance bodies [8]. There are many definitions of information technology (IT) governance.

- Among the most cited experts on the definition of IT governance are Weill and Ross, who state enterprise IT governance is “the decision rights and accountability framework for encouraging desirable behaviors in the use of IT.” They continue “IT governance is not about making specific IT decisions. That is management. Rather, governance is about systematically determining who makes each type of decision (a decision right), who has input to a decision (an input right) and how these people (or groups) are held accountable for their role.” [9, 10]
- According to CIO.com, IT governance is “putting structure around how organizations align IT strategy with business strategy, ensuring that companies stay on track to achieve their strategies and goals… it makes sure all stakeholders’ interests are taken into account and that processes provide measureable results.” [11]
- According to Gartner, IT governance “addresses two major topics: demand governance (“doing the right things”) and supply governance (“doing things right”); IT governance is the set of processes that ensure the effective and efficient use to IT in enabling an organization to achieve its goals.” [12]

Many of the above benefits and intents of enterprise IT governance likely apply to healthcare organizational imaging governance as well. Regarding specifically enterprise imaging IT governance in health care, there is no consolidated definition and no agreed upon reference white paper on the subject. Recent PubMed searches for “imaging governance,” “radiology governance,” and “enterprise governance” revealed only 46, 73, and 74 results, respectively, few of which had substantial relevance [13–15]. The HIMSS-SIIM workgroups propose the definition of enterprise imaging governance as “the decision-making body, framework, and process to oversee and develop strategies for the enterprise imaging program, technology, information, clinical use, and available financial resources.” The approach to the program, technology, information, clinical, and financial areas of focus must be carefully designed and tailored to your organization for long-term success and sustainability. The areas of focus do not imply five separate committees are needed. Rather, they suggest large subject matter areas that will need oversight in rolling out enterprise imaging. Generally, this subject matter will be incorporated as a new responsibility or new area of attention into an existing committee, or a single/few new committees will be established to undertake the responsibility. This paper will describe effective enterprise imaging governance body strategies, understanding that there is no perfect one-size-fits-all model, and that strategies will evolve as the enterprise imaging program at an institution matures.

### Program Governance

#### Intents

Like electronic health record (EHR) governance, both the enterprise imaging program governance bodies and the stakeholders should understand that the intent of governance is towards long-term strategic enablement, not erecting roadblocks. It will review short-term, occasionally myopic requests and determine sustainable, effective paths forward. Ideally, governance bodies will provide strategic requirements or guidelines for internal decision-making so that the myopic requests are not made at all. Governance should be described positively and constructively, as there are many incentives in its favor. Governance focuses attention on high impact projects and broad customer groups. Governance provides a visible group to bounce strategy off of and for stakeholders to engage with interests. Governance bodies may also provide the data security and compliance backbone to address care practices needing improvement.

**Fig. 1:**
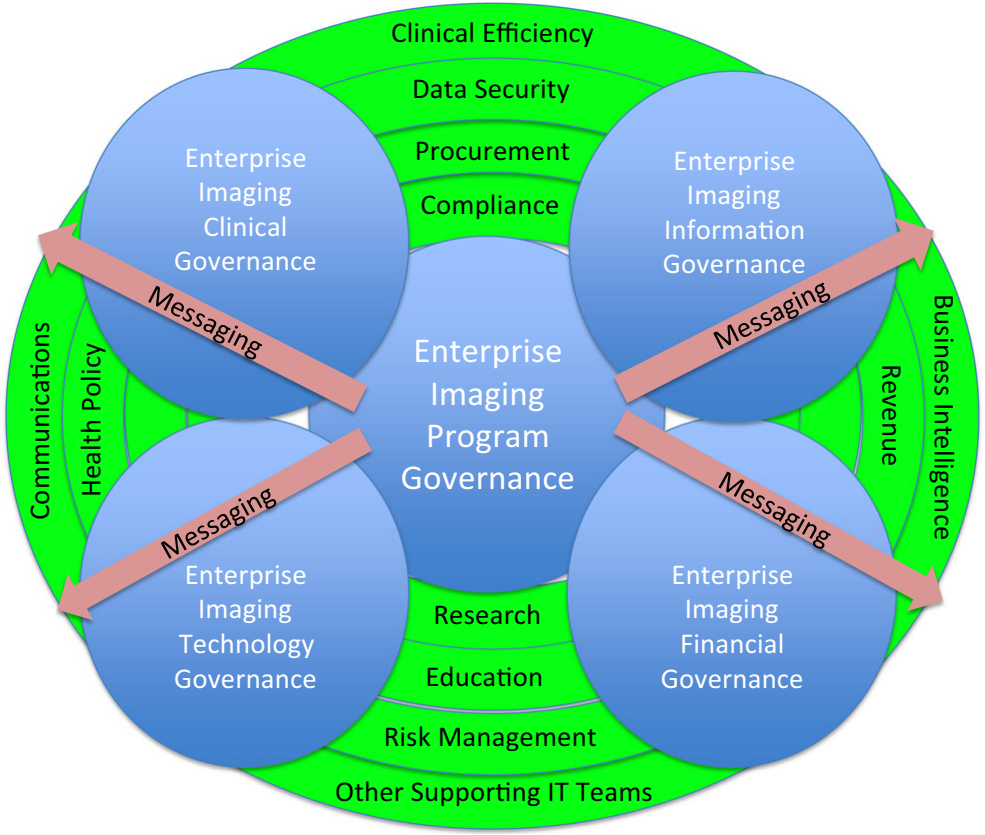
The integrated nature of enterprise imaging governance: Many non-imaging considerations impact enterprise imaging decision-making in all five areas of focus

Governance bodies should include a stated mission and charter. They should plan to build or adhere to a stated long-term enterprise imaging strategy. Governance body membership should be provided clear authority to make and communicate (often unpopular) decisions. There should be direct accountability reporting relationships between the governing body to necessary clinical and IT leadership groups and also to frontline stakeholder users [16]. How direction will be established must be known ahead of time, especially in cases where faster decisions are necessary [17–19]. Decision-making should be made and messaged out with many considerations, areas, and policies outside of imaging itself in mind (Fig. 1). The scope of the program governing body for clinical enterprise imaging should be wide, including capture, storage, distribution, viewing, and inter-facility sharing of all forms of diagnostic and possibly documentation multimedia. It must consider Digital Imaging and Communications in Medicine (DICOM) image, non-DICOM image, and video, as well as possibly scanned document, waveform, and audio data. There must be oversight to ensure clinical, research, education, revenue, compliance, business intelligence, and other needs are met. Enterprise imaging governance should touch internal medicolegal services, risk management, and credentialing departments to ensure policies and workflows are well defined and users are adhering to expectations. Similarly, the governing groups would have eyesight into external developments in health policy and in thought-leadership health informatics societies.

It is critical to define the boundaries of enterprise imaging program governance. Some enterprise imaging governance groups may be tasked with governing all aspects of imaging informatics. Some may be primarily focused on addressing imminent needs for image storage and EHR image distribution. Because imaging is so tightly integrated into clinical and department workflows, it is challenging to define where departmental governance, EHR governance, or enterprise imaging governance may have jurisdiction. Spawning of new departmental and programmatic cross-departmental questions should be expected. For example, an enterprise imaging program may discover new fluoroscopes and clinic radiography devices without systematic radiation safety management. As these modalities are discovered, questions of institutional radiation physics support may arise. Emotionally charged calls for clinical decision support for imaging modality diagnostic use may follow soon after. Similarly, virtual reality/augmented reality, imaging telehealth, 3-D printing, imaging report creation, document scanning, imaging research, and many fringe EHR considerations all are among the areas that may be in scope, out of scope, or partially in scope for enterprise imaging program governance. Unless the enterprise imaging program governance committee is specifically tasked with these considerations, program governance should recognize the importance of out of scope questions and ensure they are addressed through parallel groups.

#### Models

**Fig. 2:**
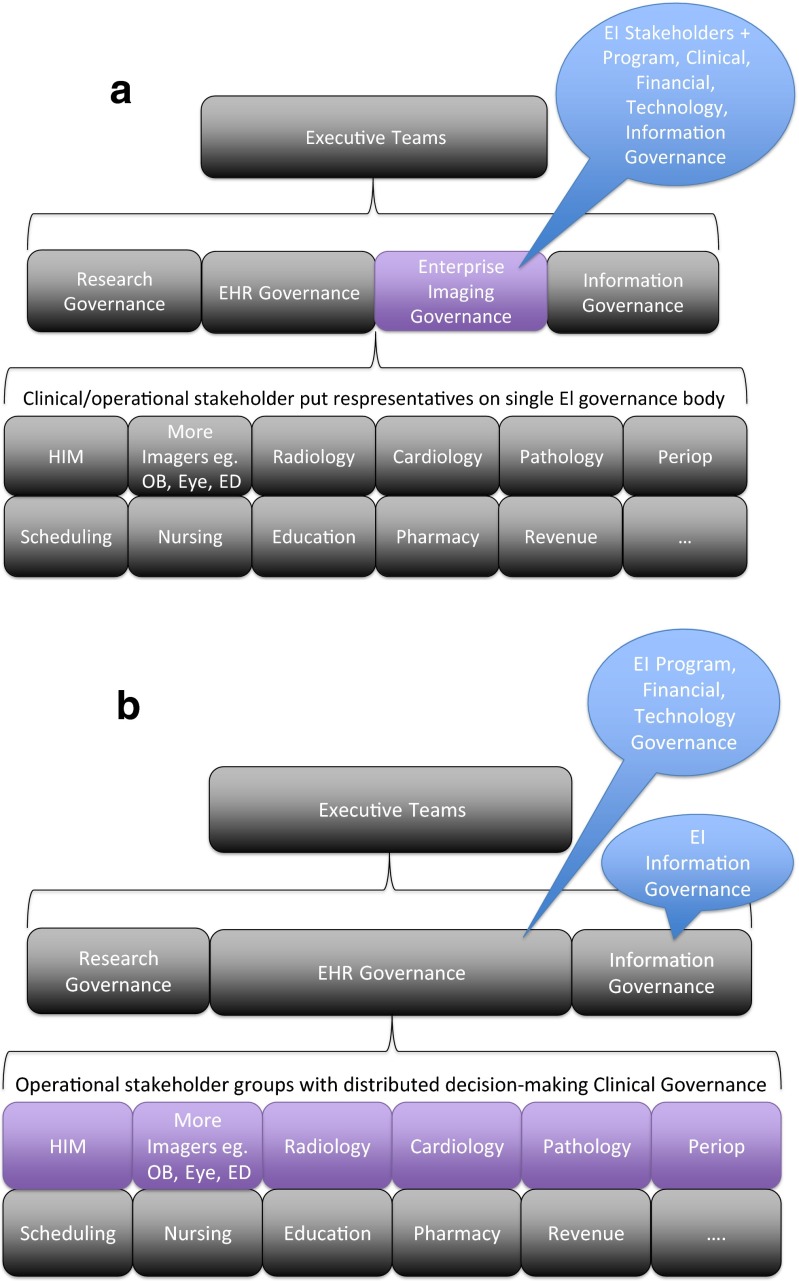
**a** Centralized Enterprise Imaging Governance Framework: Enterprise Imaging recognized as a centralized strategic group functionally separate enough to warrant its own group governing body with imaging heavy Chairs and Vice Chairs and informatics leadership such as CMIO/CIO. Most decisions for enterprise imaging made in a single council. *EI* Enterprise Imaging. **b** Distributed Enterprise Imaging Governance Framework: Enterprise Imaging primarily considered an integrated aspect of the EHR. Stakeholder groups with larger flexibility to innovate and design solutions within a set of guidelines set by EHR Governance. *EI* Enterprise Imaging

When considering the five areas of focus of enterprise imaging governance, program governance tends to exist at the highest level of the five within the organization, commonly within the highest levels of electronic health record governance, or having a parallel committee at the same level. After that, different enterprises distribute decision-making power differently depending on size, strategic goals, existing governance model, and the above described boundaries expected of the enterprise imaging governance group [20]. Different models of distributing the five areas of focus of enterprise imaging governance may be considered (Fig. 2a, b). In a centralized model (Fig. 2a), a single enterprise imaging steering committee makes the majority of governance decisions. The central committee centralizes image management decision-making around a group of leaders closest to enterprise strategic development, necessary financial approvals and inherently brings program awareness. In event of an institutional merger, acquisition, or divestiture, the enterprise imaging program will be well positioned to transition easily. While being empowered to drive steps of planning and implementation, the leadership individuals on this committee however may be less familiar with specific needs, necessary technologies, and may not be practicing clinical users of the enterprise imaging systems. It is important with this “top down” program governance approach for the implementation teams to strongly engage clinical front line stakeholders to confirm that workflow and infrastructure decisions made by program leadership are usable. In a distributed model (Fig. 2b), hospitals incorporate program governance both “top down” by leadership as described and also strongly encourage “inside out” governance if very effective EHR governance already exists at that site. “Inside out” program governance can bring many clinical governance decisions within groups closest to and most knowledgeable about local workflow and user preferences within a set of guidelines determined by program governance informatics and clinical leaders. EHR stakeholder committees understand user pain points and know the limitations of each downstream system. Using the distributed model inherently requires greater communication. Whether enterprises choose to govern imaging more centralized or more distributed, the necessary intents of governance towards overseeing strategy, purchasing power, prioritization, resourcing, et cetera must be clearly established.

#### Culture and Makeup

Committee member roles and responsibilities should be clearly defined. The reporting relationships for personnel supporting the enterprise imaging endeavor are critically important for the program committee for fair optics. Its membership should be inclusive of leaders representing technology, information, provider and non-provider clinical, and financial governance. Successful enterprise imaging program governance requires alignment between clinical operations senior leadership, IT senior leadership, and IT operational team representation. The governing body should serve as an executive forum for relationship building and knowledge sharing between imaging service lines and leaders. Governance bodies guiding the enterprise imaging program should be department or service line agnostic. To that end, the program governing body must reassure all specialties involved through the governance charter that the intent is not to take imaging capabilities away or even to necessarily consolidate services. Instead program governance should act to balance individual clinic and user needs against defined enterprise informatics strategies, widespread ease of use, and reasonable build maintenance.

Groups finally sitting at the program governance table together equally also may not have a great working relationship to that point; getting spine orthopedic surgeons together with neurosurgeons, or imaging-minded cardiologists together with cardiothoracic and vascular radiologists might require a deft touch and an understanding of any preexisting hospital politics. It is important to recognize that each clinical specialty has its own imaging expertise, though the governance body will learn that there is a broad range of maturity today in their ambulatory and acute health care image management. Specialties like radiology and often cardiology will have decades of successful image management expertise and have deeply integrated imaging into the care they provide. These specialties have technologist staff certifications and provider residency board exams that test image capture and management best practices. These specialties may be unnerved by having a single seat at the enterprise table next to more imaging immature specialties. Radiology and cardiology can serve as leaders of such a governance body and as mature operational models for less experienced specialties to pattern after. Radiology and cardiology should not expect other specialties to simply follow their time-tested workflows however, as the workflows will fail in many other locations. Specialties in whom image management is only beginning to emerge, such as telemedicine, telepresence, and pathology can learn select best practices quickly with this model; these specialties in fact often are appreciative of not having to figure out new imaging workflows themselves and can lean on enterprise governance requirements to drive user adoption. Other specialties, such as endoscopy procedure, radiation oncology, dermatology, and wound care may also have years of image management expertise, but only in a very narrow set of workflows and care use cases. These groups often come into enterprise imaging needing standardization of user workflows rather than education and will benefit from the enterprise imaging focus as mature infrastructure is finally made available to them. Enterprise content management of scanned documents, reports, and results may also require representation on the program governing body if the storage hardware is to incorporate these objects as well. Given the range of maturity levels, use cases, and resources available in each specialty, it is imperative to foster a culture of respect and collaboration in the governance group.

### Technology Governance

Enterprise imaging technology governance bodies may include EHR analysts, PACS/archiving analysts, modality engineering, interfaces analysts, business continuity and disaster recovery analysts, project management, the enterprise help desk, as well as provider informatics leadership and operations. Appropriate technical oversight governance must weigh the capabilities of existing enterprise DICOM brokers, DICOM modality worklist tools, wired and wireless network bandwidth capacity, and existing and future interface initiatives. Technology governance also must consider routine daily infrastructure utilization when planning data migrations and ongoing IT staff activity. Technology governance group decision-making must weigh the costs and benefits between potential primary and backup image storage architectures, local storage versus off-site cloud based offerings, and the associated disaster recovery and/or business continuance implications. For example, many academic centers are interested in saving images “forever” rather than employ image lifecycle management driven purging of minimally valuable imaging beyond mandating storage periods. Saving imaging data for long periods must account for hardware and software obsolescence, as often more than one data storage medium change or data center migration will occur during the retention period of a given pediatric imaging case. Technology and/or information governance groups must determine imaging record, image retention, image lifecycle processes, and long-term image compression based on not only what is appropriate for the cost, setting, image type, use case, user base being considered but also 42 CFR 482.26(d) federal requirements [21], state requirements [22], local statute of limitations, relevant society expectations, and best practices [23–26].

While hardware, software, and knowledge investments in enterprise imaging will be required, there will be long term operational cost offsets that governing groups might expect [27]. These offsets include management of fewer standalone DICOM storages and fewer clinical and IT staff necessary for imaging support. Vendor support costs also grow more favorable over time with progressive storage and viewing application consolidation. In pursuing enterprise imaging, governance processes must commit procurement and clinical business units to current and potentially legacy hardware standardization. Often, existing clinical imaging acquisition modalities will be found technically insufficient to send images to a third party archive at the time the program begins; legacy modalities purchased years ago without the expectation of saving images to an EHR often do not have short-term image storage capabilities, purchased vendor licenses for DICOM features, or wireless network capabilities. This provides an opportunity for governance to educate users on the requirements for enterprise imaging and to insist on moving to more homogeneous imaging hardware through retrofitting or purchase. During these education and requirement conversations, it is often time effective to discuss controlled and funded projects for innovation. In general, enterprises should expect that imaging technology governance be made aware and involved early as new projects are considered.

### Information Governance

The American Medical Informatics Association (AMIA) acknowledges widespread agreement on the high value of secondary data use. AMIA encourages the data be as consistent, comparable, timely, accurate, accessible, complete, and reliable as possible [28]. The American Health Information Management Association (AHIMA) defines information governance as the “enterprise-wide framework for managing information throughout its lifecycle and supporting the organization’s strategy, operations, regulatory, legal, risk, and environmental requirements” [29, 30]. AHIMA states that information governance ensures that information is trustworthy and actionable through alignment with organizational strategy and engagement of senior leaders for the purposes of leveraging it as a strategic asset for organizational decision-making, performance improvement, cost management, and risk mitigation [31, 32]. A study by AHIMA confirmed that most healthcare organizations have only recently developed information governance groups and frameworks [30]. Strong and systematic information governance for imaging specifically in most healthcare organizations is generally felt by the HIMSS-SIIM collaborative group to be new as well.

Images, image metadata, video, and audio files as clinically necessary data, information, and knowledge must be acknowledged and supported by the highest levels of enterprise governance. Governance processes may be necessary to enforce the policies and workflows that ensure mandatory, conditional, and optional imaging metadata capture at the point of care. Information governance must define both what image data and image metadata will be made available for data warehousing and analytics, and how this data will be accessed. The data and metadata may be unfamiliar to many non-imagers around the organization. Information governance may require association of imaging metadata to RadLex/LOINC, SNOMED, CPT, and ICD-10 codes. It should consider not only EHR exam timestamp but also modality DICOM timestamp capture.

Information governance grows in importance as imaging data and metadata re-use expands. [33–36]. There are many needs and use cases involving image data and metadata re-use for information governance to consider. Practical clinical and research applications such as in machine learning algorithms towards computer vision and anatomic segmentation are being developed. Educational re-use such as in teaching file creation is also increasingly prevalent. With this growth, raw medical image information is increasingly valuable to an organization and its distribution must be well controlled. Governance of secondary imaging dataset use must consider appropriate patient data protection and informed consent to use. De-identification and anonymization of many clinical imaging datasets can be challenging, especially if they include “burned in” pixel data often commonly found in video content, secondary capture images, or ultrasound images.

The lines between enterprise image management and enterprise content management, together often described as enterprise clinical multimedia content, continue to blur. Information governance relationships between enterprise imaging teams and enterprise health information management (if they are separate at all) should be fostered and deepened. Technical design, hardware and clinical workflow may overlap significantly between the two entities. Electronic health record support teams will be required to ensure metadata content ownership is assigned appropriately and the multimedia object content being integrated best informs the patient record alongside text-based content.

Pro-active awareness of image management law and regulatory changes fall into expected scope for information governance [37]. Information privacy and security risks must be addressed comprehensively through governance policies involving appropriate use, enterprise data warehouse audit procedures, device encryption, adequate authentication practices, and other means. Such policies for imaging include handheld camera images of child abuse, plastic surgery, and other sensitive electronic information.

As in any large healthcare program or initiative, enterprise imaging requires establishing key metrics for success, such as cost reductions in hardware, portable storage media, and personnel effort; clinical care impact; more secure data; improved hospital compliance; research; education; even medicolegal and public media risk aversion. Program governance groups typically own the metrics selection and report out, while information governance is responsible for the data gathering and collating. Information governance will be closely involved with cross specialty imaging nomenclature definition, as well as governing provider level and patient level dashboarding applications.

### Clinical Governance

In the early stages of an enterprise imaging project, clinical team members are often unaware of the broad spectrum of specialties performing imaging at their facility, [38] or that many departments and areas share the same image management pain points they personally experience day to day [39, 40]. Provider-led governance bodies and provider champions can be vital to explain the clinical and non-clinical business cases for delivering easily consumable images to the masses. While many providers will view an enterprise imaging endeavor as value added, others will focus on any additional mouse clicks and resist the additional effort. The latter group includes those providers who may be less familiar with image capture and image management best practices, may have less experience having the benefits of prior images available, or may see the effort as an extension of a frustrating electronic health record project. While an IT-oriented governance body may highlight enterprise imaging as leveraging the large investments made in enterprise EHRs and trumpet institution-wide savings on maintenance and support costs, such reasonings may be less tangible to the revenue-generating individual clinical users. A clinical imaging governance group must define such tangible wins. One such tangible win for providers would be vetting, selection, and implementation of an enterprise media viewer; [41] a largely provider-led clinical enterprise imaging governance is the ideal group to pursue this goal. Other tangible wins to describe could include a more image-enriched patient record and easier cross-facility image sharing in busy provider clinics and hospitals [42]. Multi-disciplinary conferences would more easily include all forms of salient medical information. Non-clinical benefits important to providers and clinics are equally important to detail, including offering revenue justification, fulfilling Meaningful Use menu objectives and accreditation body requirements, occasionally supporting provider medicolegal inquiries, and the ease of image acquisition for education and research purposes.

Building an enterprise imaging governance body takes clinical and non-clinical staff time. Enlisting provider involvement on clinical enterprise imaging governance committees in particular can be a challenge. Some entities more mature in enterprise imaging have provided administrative salary support/supplementation and/or leadership positions to practicing provider champions. The amount of physician and per-physician dedicated time allotments will vary per organization and depends on the local program governance model. Having such dedicated time for enterprise imaging however places accountability on physicians to lead project expansion, makes more likely that metadata captured will have clinical purpose and eases engaging less interested physicians by adding cache to the initiative.

### Financial Governance

Financial governance as a focus area may be easily folded into “top down” program and even technology governance at many institutions. In the early stages of an enterprise imaging initiative, prudent financial governance must be exercised around capital acquisition and vendor management, operating budgets, personnel and human resources, and a balanced model for support. This financial governance is best from the IT C-suite, the business C-suite, and potentially from Chairs and Vice Chairs of high powered imaging departments like radiology and cardiology after a thorough evaluation of image storage, viewing, and sharing needs across the enterprise.

A core responsibility of early enterprise imaging program and financial governance is assistance with prioritization of work projects. Program and/or financial governance would direct teams towards highest yield projects. An institution may prioritize projects where there may be compliance concerns (necessary images are not systematically kept) or patient safety concerns (ongoing review and monitoring of local radiation producing hardware is necessary). Alternatively, an institution may look to recover money paid for enterprise imaging infrastructure and prioritize projects with a large cost avoidance (decommissioning expensive storages and viewers) or a hard dollar reimbursement win (imaging telemedicine growth or better charge capture for point of care ultrasound). Some governance bodies may attempt a scoring rubric to systematically and fairly rank imaging sites with the most favorable business cases. Such scoring rubrics may include not only costs and revenue generated but also non-financial characteristics such as impacts on training, user workflow, research, patient satisfaction, patient safety, and regulatory compliance. As new areas are being considered for inclusion into the enterprise imaging architecture, strong financial governance should require definable return on investment (ROI), return on health (ROH), and deployment cost evaluations for each, with report out through program governance.

Financial governance groups may assign total costs in enterprise imaging in several ways. An *a la carte* cost assignment of costs based on number of exams, average data requirements per exam, and/or FTE headcount could be made fair and straightforward. This is similar to the historical model of departmental funding of infrastructure and storage capacity in place at many organizations. This model, however, could be cost prohibitive to specialties like pathology whose data requirements may soon dwarf radiology and cardiology combined, who do not have proportionally larger top line revenues, and who may not make additional revenue by capturing images. Such a financial model also provides ammunition for more resistant providers to push back on systematic image storage. Alternatively, human and infrastructure costs for enterprise imaging may be rolled into enterprise EHR costs. Since images are captured in routine clinical care and documented in routine clinical EHR notes, enterprise imaging could simply be defined as the cost of doing business today, with costs buried and allocated to the health organization together. In this model, some specialties may cry foul because a power user specialty like radiology generates more data and requires more FTEs for support than others but may not bear a higher proportion of the cost. Noting, however, that specialties all use related EHR functionality differently and save varied amounts of data to the EHR; this argument may not be valid to a financial governance group. Decisions on the cost attribution model and calculations for ROI and ROH used will likely be very locally driven.

## Conclusion

This HIMSS-SIIM white paper highlights the need for programmatic governance body oversight of enterprise imaging technology, information, clinical use, and financial impacts. The governance models will vary between organizations based on their size, geographic distribution, current image storage and distribution technology, and the breadth of specialties practiced at the facility. Since enterprise imaging is inherently a horizontal initiative spanning multiple clinical verticals, governance representation should be similarly broad. In this way, effective enterprise imaging governance may require as much provider and non-provider documentation governance as it does diagnostic imaging specialty governance. The governance body or bodies should involve many provider and non-provider users and administrators. Enterprise governance leadership must assist with setting scope decisions, reasonable expectations for rollout, and the prioritization roadmap. The governance processes should be transparent and quick to respond to new requests or environmental changes. Only with strong enterprise imaging governance will the enterprise imaging program be successful.
